# Supplementary figures and images for: MR Relaxometry for Discriminating Malignant Ovarian Cystic Tumors: A Prospective Multicenter Cohort Study
Source: Diagnostics (Basel). 2024 May 21;14(11):1069. doi: 10.3390/diagnostics14111069 (PMC11172376; doi:10.3390/diagnostics14111069)

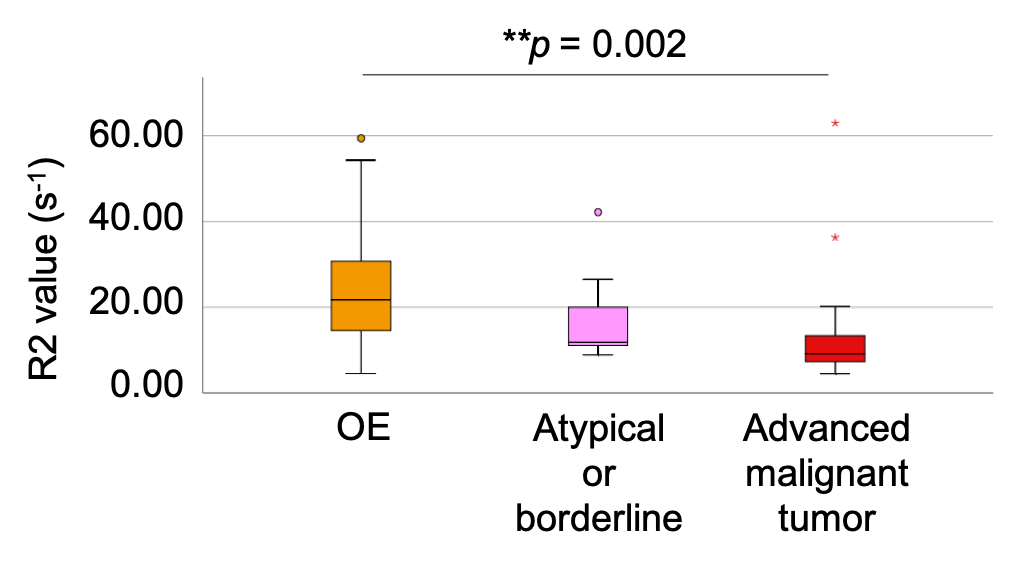

Supplement: Supplementary file 1 [file diagnostics-14-01069-s001.zip › diagnostics-2976594-supplementary.tif]
